# A singleton pregnancy with placental chorioangioma and hydrops fetalis complicated with mirror syndrome and ritodrine-induced side effects: a case report

**DOI:** 10.1186/s12884-024-06391-5

**Published:** 2024-03-20

**Authors:** Pei-Tzu Wu, Kun-Long Huang, Ching-Chang Tsai, Hsin-Hsin Cheng, Yun-Ju Lai, Te-Yao Hsu

**Affiliations:** Department of Obstetrics and Gynecology, Kaohsiung Chang Gung Memorial Hospital, Chang Gung University College of Medicine, 123, Ta-Pei Road, Niao Sung District, Kaohsiung, 833 Taiwan

**Keywords:** Ritodrine hydrochloride, Rhabdomyolysis, Mirror syndrome., Placenta chorioangioma, Hydrops fetalis, Acute liver injury, Acute kidney injury

## Abstract

**Background:**

Ritodrine hydrochloride is a widely used beta-adrenergic agonist used to stop preterm labor in Taiwan. Many side effects causing maternal morbidity and mortality have been reported. We report a case complicated with ritodrine-induced side effects and mirror syndrome that was associated with placental chorioangioma.

**Case presentation:**

A 36-year-old singleton pregnant woman at 25 6/7 weeks of gestation, with an undiagnosed placental chorioangioma, underwent tocolysis due to preterm uterine contractions. Her clinical condition deteriorated, attributed to mirror syndrome and adverse events induced by ritodrine. An emergency cesarean section was performed at 27 1/7 weeks of gestation, delivering an infant with generalized subcutaneous edema. A placental tumor measuring 8.5 cm was discovered during the operation, and pathology confirmed chorioangioma. Gradual improvement in her symptoms and laboratory data was observed during the postpartum period. Identifying mirror syndrome and ritodrine-induced side effects poses challenges. Therefore, this case is educational and warrants discussion.

**Conclusion:**

Our case demonstrates mirror syndrome induced by chorioangioma, which is rare, and ritodrine-induced side effects. The cessation of intravenous ritodrine and delivery are the best methods to treat maternal critical status due to fluid overload.

## Background

Ritodrine hydrochloride is a beta-2-adrenergic agonist. It is commonly used for tocolysis, but numerous adverse effects have been reported, including tachycardia, pulmonary edema, cardiomyopathy, hyperglycemia, parotid pain, psychiatric symptoms and agranulocytosis. Rhabdomyolysis and acute liver injury have also been described in some patients with ritodrine hydrochloride administration [1–7]. .

Ritodrine has been removed from US market according to FDA announcement. The European Medicines Agency’s Pharmacovigilance Risk Assessment Committee has similarly stated that prolonged use of high-dose short-acting beta-agonists for obstetric indications poses a serious risk of cardiovascular side effects to both the mother and fetus. Consequently, the use of this medication is prohibited in the European Union and the United States. Ritodrine hydrochloride was the most widely used beta-adrenergic agonist to stop preterm labor in Taiwan before 2022 because of the policy of the Taiwanese National Health Insurance, which suggests that intravenous ritodrine be used as the first-line treatment for tocolysis. Here, we present a case of a singleton pregnancy complicated with multiple ritodrine-induced side effects, including rare diagnosis of rhabdomyolysis. Initially, we did not realize the diagnoses of maternal mirror syndrome induced by chorioangioma and hydrops fetalis until the placental chorioangioma was found during cesarean section. It is difficult to determine whether mirror syndrome or ritodrine was the major cause of maternal pulmonary edema. Thus, this rare case is worthy of discussion due to the rare complications and associated placental disease.

## Case presentation

A 36-year-old Taiwanese female patient, gravida 2, para 1, with a previous vaginal delivery history and without a medical or surgical history was initially admitted to a private hospital due to preterm uterine contractions at 25 weeks and 6 days. Continuous intravenous infusion of ritodrine hydrochloride at the dose of 6 mg/hour was given. After receiving tocolytics for one week, preterm uterine contractions with vaginal bleeding persisted, so the patient was transferred to the emergency department of Kaohsiung Chang Gung Memorial Hospital.

At triage, the patient’s vital signs were as follows: a body temperature of 37.2 °C, a pulse rate of 133 beats/min, a respiratory rate of 16 breaths/min, and a blood pressure of 120/80 mmHg. Laboratory tests revealed the following: acute kidney injury (serum creatinine level of 3.94 mg/dL), hyponatremia (serum sodium level of 120 mEq/L), hyperuricemia (serum uric acid level of 13.2 mg/dL) and elevated levels of liver enzymes (serum alanine aminotransferase (ALT) level of 202 U/L and serum aspartate aminotransferase (AST) level of 192 U/L). The urine analysis was normal. The patient did not initially present with dyspnea at our emergency department. Tocodynamometry revealed regular uterine contractions, and the fetal heart rate was reactive. The cervix, which was dilated to 3 cm, had bloody tissue. Thus, the patient was admitted to our tocolytic unit due to preterm labor, acute kidney injury and impaired liver function.

**Fig. 1:**
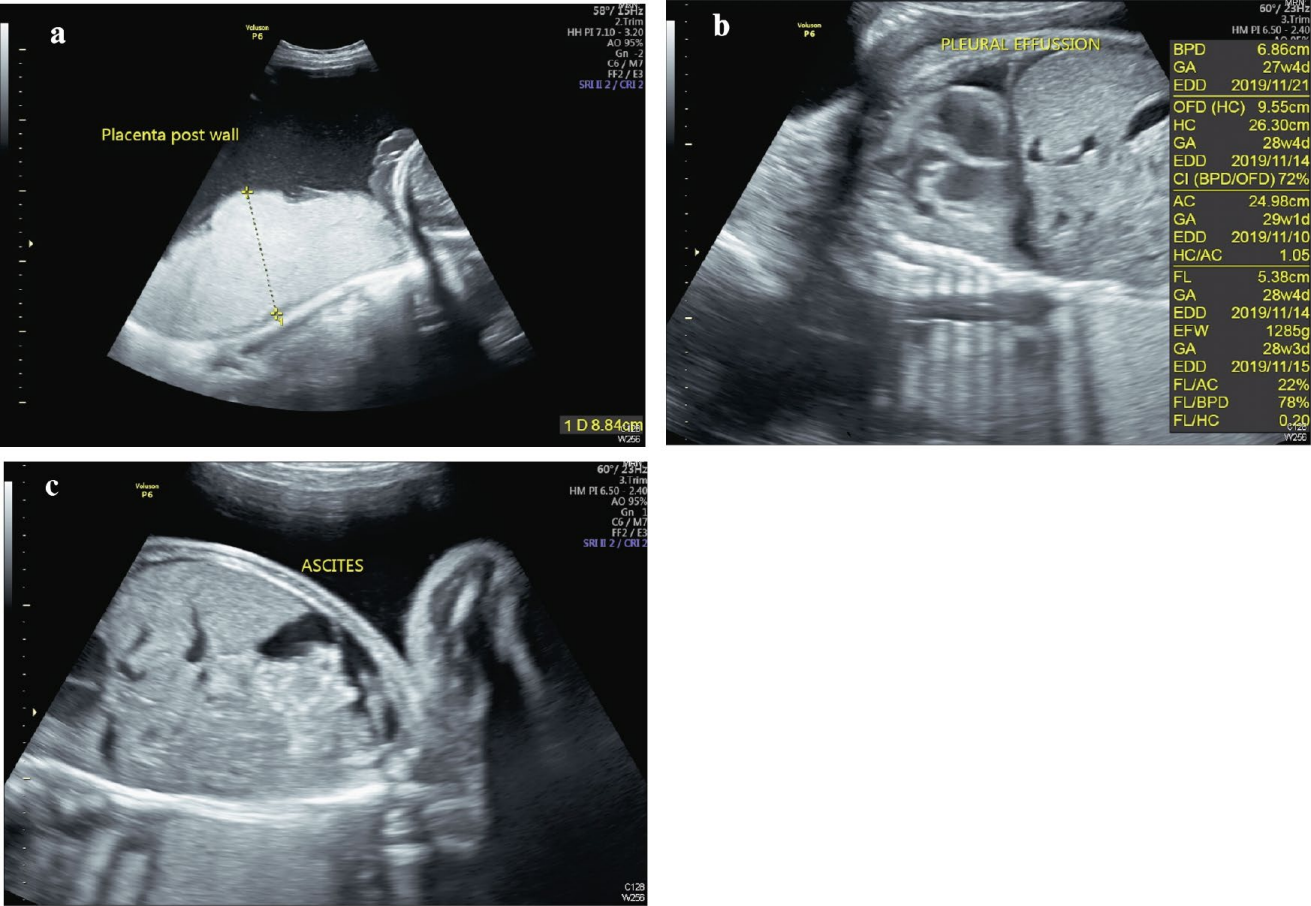
**A** Transabdominal sonography showing the placenta over the posterior wall of the uterine cavity with increased thickness (approximately 8.8 cm). **B** Transabdominal sonography showing fluid accumulation in the bilateral pleural cavity of the fetus. **C** Transabdominal sonography showing fluid accumulation in the abdominal cavity of the fetus. According to the findings presented in Fig. 1B and -C, hydrops fetalis was diagnosed

**Fig. 2:**
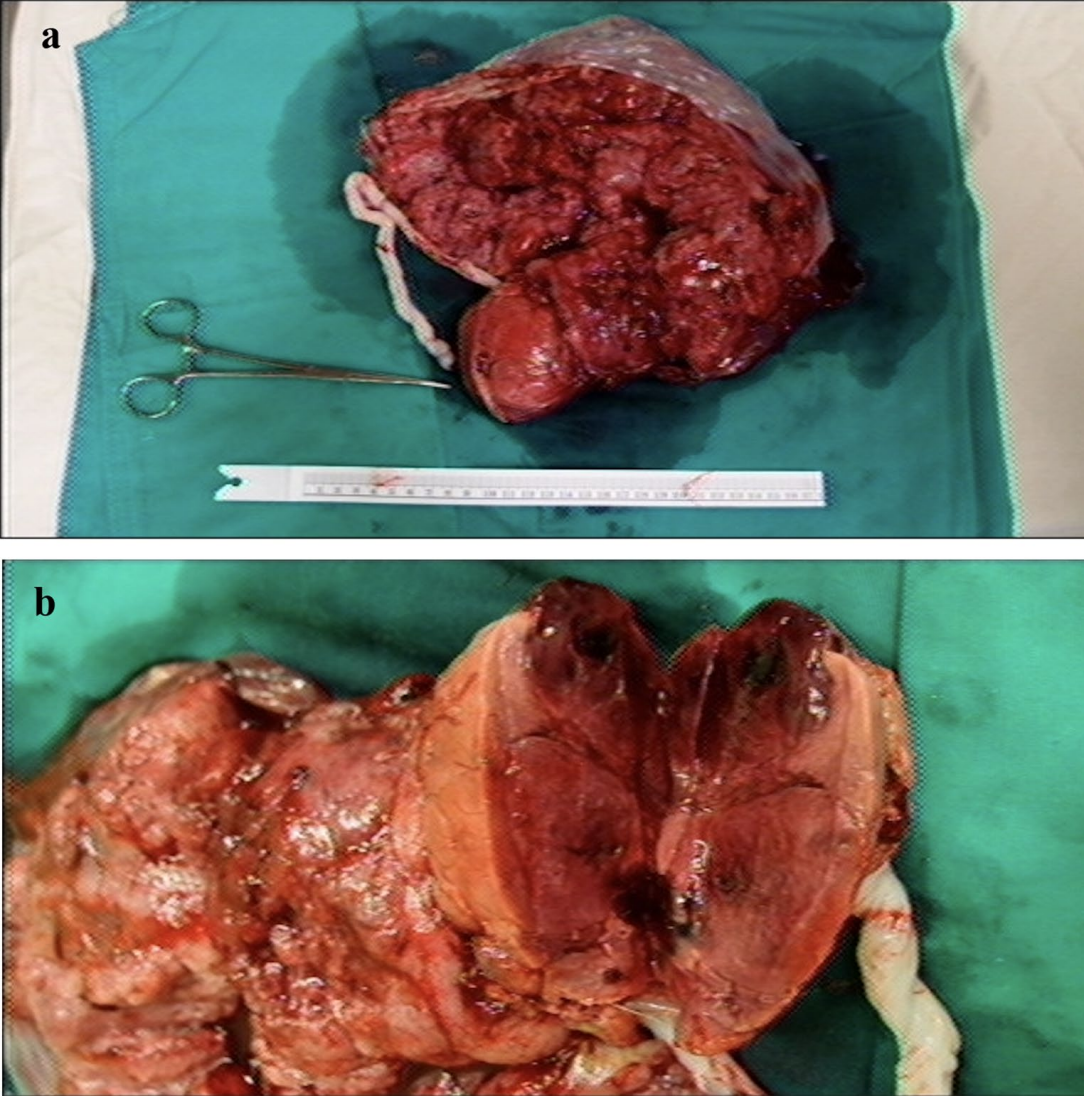
**A** After opening the placental membrane and exposing the placental tissue, an 8.5-cm encapsulated tumor was observed in the periphery of the placenta. **B** After excision, the placental tumor was observed to have firm and congested solid content. The pathology was chorioangioma

After admission, atosiban acetate, which is an antagonist of the oxytocin receptor, was given intravenously at a rate of 100 µg/min in combination with ritodrine to complete the course of intramuscular betamethasone and intravenous magnesium sulfate. Due to impaired renal function, we administered a standard loading dose of 4 g magnesium sulfate for neuroprotection without a maintenance dose. Ritodrine was maintained at a dose of 6 mg/hour through an intravenous line. However, the patient presented with dyspnea, symmetric bilateral pitting edema of the lower limbs (grade 2) and dark urine two days after admission. Chest radiography revealed bilateral pulmonary edema. Electrocardiography excluded acute myocardial infarction. The follow-up laboratory findings revealed progressive acute kidney injury (serum creatinine level of 4.13 mg/dL) and persistent impaired liver function (serum AST level of 126 U/L and serum ALT level of 82 U/L) with hypoalbuminemia, progressive hyponatremia (serum sodium level of 116 mEq/L), hypermagnesemia (serum magnesium level of 5.6 mEq/L), and hyperuricemia (serum uric acid level of 14.6 mg/dL). The arterial blood gas data showed high anion gap metabolic acidosis. The serum creatinine kinase (CK) level was elevated at 1202 U/L. Ritodrine was ceased before cesarean section. Transabdominal sonography showed polyhydramnios with an amniotic fluid index of 40 cm, placental thickening (88 mm on the posterior wall of the uterus, Fig. 1A), and hydrops fetalis (Fig. 1B and -C). Because of the deteriorated maternal and fetal status, emergency cesarean section was performed at 27 weeks and 1 day of gestation. A 1000-g female infant was delivered and had an Apgar score of 1 at 1 min and 3 at 5 min. After cardiopulmonary cerebral resuscitation and intubation, the neonate was transferred to our neonatal intensive care unit for respiratory distress syndrome, bronchopulmonary dysplasia and necrotizing enterocolitis. A placental chorioangioma (8.5 × 6.5 × 4.2 cm in size) was found after delivery of the placenta (Fig. 2A and B). The histopathological analysis indicated that the tumor consisted of proliferative capillaries within a variably cellular and collagenous stroma, confirming the diagnosis of placental chorioangioma. At this time, we realized that hydrops fetalis, polyhydramnios, placental thickening and maternal mirror syndrome were induced by the placental chorioangioma.

The patient was transferred to the intensive care unit after cesarean section. She was treated with sodium bicarbonate, adequate fluid hydration, and albumin supplementation. Her clinical status and laboratory indicators improved gradually after management. The patient was extubated and transferred back to the ordinary ward three days later. The patient was discharged home on the seventh day after cesarean section. The newborn was discharged home three months later and later developed complications, including hyperactivity and obsessive-compulsive disorder at four years old.

## Discussion and conclusions

### Placental chorioangioma and maternal mirror syndrome

Chorioangioma is the most common benign nontrophoblastic vascular tumor of the placenta and occurs in approximately 1% of pregnancies [8]. Most chorioangiomas are small and do not cause symptoms. The incidence of large chorioangiomas, defined as more than 4–5 cm in size, varies from 1:3500 to 1:9000 (0.29–0.11%) [9]. These larger tumors may cause maternal complications and adverse prenatal fetal outcomes, including polyhydramnios, maternal preeclampsia, maternal mirror syndrome, preterm delivery, fetal heart failure, fetal anemia and thrombocytopenia, fetal growth retardation, hydrops fetalis, fetal demise, severe neonatal microangiopathic hemolytic anemia, thrombocytopenia and neonatal death [8, 10]. The pathophysiological mechanisms of these complications are unclear, but a hypothesis that can explain these adverse outcomes is chronic arteriovenous shunting, which sequesters red blood cells and platelets within the tumor and develops into high-output fetal cardiac failure [8]. D Buca et al. also summarized that the risk may increase with increasing tumor size and that the prevalence of these adverse outcomes may be higher if the fetus presents with fetal hydrops [10].

Mirror syndrome (MS) is defined as the development of maternal edema combined with fetal hydrops. Despite the unclear pathogenesis and prevalence of MS, it is associated with rhesus iso-immunization (29%), twin-twin transfusion syndrome (18%), viral infection (16%), fetal congenital anomalies, and fetal or placental tumors (37.5%). There are several clinical manifestations that may be present in MS patients, such as maternal edema, headache, visual disturbances, elevated blood pressure, mild anemia with hemodilution, a low platelet count, elevated serum levels of liver enzymes, uric acid, and creatinine, oliguria, albuminemia and proteinuria [11]. Nevertheless, this disorder is often misdiagnosed because of preeclampsia-like manifestations. Differentiating between MS and preeclampsia-like syndromes may be difficult, and the only difference lies in fetal edema, which is present in MS [12, 13]. Our case demonstrated a larger chorioangioma, which was 8.5 cm in diameter, and the patient presented with severe pulmonary edema, progressive lower leg edema and hydrops fetalis. These findings indicate that a larger tumor size is associated with more maternal and fetal complications [14]. We also summarized recent similar cases with chorioangioma in Table 1 from the literatures [14, 15]. All cases had chorioangiomas measuring more than 6 cm, and the gestation weeks ranged from 27 to 29. Maternal complications included polyhydramnios, preterm prelabor rupture of membrane, mirror syndrome and chorioamnionitis. The most common fetal complication was preterm birth. Among the three cases treated with ritodrine, two cases (case 2 and 4) resulted in stillbirth during labor.

**Table 1:** Maternal-fetal complications and pregnancy outcomes among cases with chorioangioma similar to ours

Reference	Case	Tumor diameter (cm)	Maternal complications	Fetal complications	Ritodrine use	GA at delivery (weeks)	Pregnancy Outcome
Our case	1	8.5	Mirror syndrome Polyhydramnios	Fetal hydrops Preterm birth	Y	27 1/7	NICU admission, NRDS, NE, BPD
Ma et al. (2023)	2	10	Polyhydramnios PPROM	Preterm birth	Y	28 4/7	Stillbirth during labor
	3	8	Polyhydramnios PPROM	Fetal hydrops Fetal anemia Preterm birth	U	28 4/7	NICU admission NIH
	4	7	Polyhydramnios	Preterm birth	Y	27 2/7	Stillbirth during labor
	5	6.5	Polyhydramnios	Preterm birth	U	29 4/7	NICU admission, NIH, BPD
	6	6	Polyhydramnios PPROM Chorioamnionitis	Fetal anemia Preterm birth	U	29 3/7	NICU admission, NRDS, NIH, BPD Neonatal shock Neonatal death
García-Díaz et al. (2012)	7	7.8	Mirror syndrome Polyhydramnios	Fetal anemia Mild cardiomegaly Fetal hydrothorax	N	29 5/7	Neonatal death

GA: gestational age, PPROM: preterm prelabor rupture of membrane, NICU: Neonatal intensive care unit, NRDS: Neonatal respiratory distress syndrome, NE: Necrotizing enterocolitis, NIH: Neonatal intracranial hemorrhage, BPD: Bronchopulmonary dysplasia, Y: use of ritodrine, U: unknown, N: without ritodrine use

We considered the chorioangioma to be the major cause of maternal MS because the patient did not initially present with these symptoms after seven days of intravenous ritodrine treatment at another hospital. However, ritodrine hydrochloride may have been the aggravating factor that induced MS. At our hospital, we did not find out the placental tumor at the first time, so continuous tocolysis was given. Although we performed repeated ultrasound examination when hydrops was seen, we still missed the large placental tumor. Authors thought that we should pay more attention to those diseases associated with hydrops fetalis during ultrasound examination. Besides, early delivery and the cessation of tocolysis are the best management strategies for patients with MS and rare side effects of ritodrine. Therefore, we presented this case to alert physicians to be vigilant about this type of maternal-fetal conditions.

### Association between ritodrine-induced side effects and chorioangioma

Many ritodrine-induced side effects have been documented. Cardiovascular adverse events are linked to the stimulation of beta-1 and beta-2 adrenergic receptors. Symptoms may include tremors, palpitations, shortness of breath, chest discomfort, and pulmonary edema [16, 17]. Ritodrine may lead to metabolic effects, such as hypokalemia and hyperglycemia. The effects on the fetus are similar to those on the mother, but prolonged maternal hyperglycemia may lead to neonatal hyperinsulinemia and, consequently, hypoglycemia [16]. However, there is limited evidence of the association between ritodrine-induced side effects and placental chorioangiomas. Based on our case and findings, we posit that the side effects of ritodrine may be heightened and exacerbated by chorioangioma due to the maternal fluid-overloaded status.

### Ritodrine-induced rhabdomyolysis

Rhabdomyolysis is a complex medical condition characterized by the dissolution of damaged muscle cells, which results in the release of intracellular contents, including electrolytes, myoglobin, and other sarcoplasmic proteins, such as creatine kinase, lactate dehydrogenase, alanine aminotransferase, and aspartate aminotransferase, into the bloodstream. Both traumatic and nontraumatic rhabdomyolysis may manifest as limb weakness, myalgia, swelling, and gross pigmenturia without hematuria [18]. Rhabdomyolysis can be induced by genetic defects, muscle hypoxia, infections, body-temperature changes, metabolic disturbances, or drugs and toxins, any of which may result in multiple organ impairment due to the secretion of myoglobin into the blood circulation. Acute kidney injury may be complicated by severe rhabdomyolysis, which is thought to be a major factor determining prognosis [7]. The mechanism of ritodrine-induced rhabdomyolysis is still unclear, and three possible potential mechanisms have been reported: (1) Direct drug damage caused by administering ritodrine in rats [19]; (2) Hypokalemia, which leads to muscular necrosis [20]; and (3) Coexisting muscular disorders, such as dystrophia myotonica (DM), which makes an individual more susceptible to side effects of ritodrine [21, 22]. Ritodrine-induced rhabdomyolysis associated with DM has a shorter onset interval between the administration of ritodrine and symptom manifestation [23]. In our case, the patient had been tested for DM genes, and the results of both cytosine-thymine-guanine and cytosine-cytosine-thymine-guanine expansion repeats were within the normal range.

Matsuda et al. concluded that after persistent use of tocolytic therapy for more than a week, approximately a quarter of patients may present elevated CK levels above the normal range. The increased in CK levels is correlated with the total dose of tocolytics, especially in patients with concomitant use of both ritodrine and magnesium sulfate [24]. Serum CK levels beyond five times the upper limit of normal are often used for diagnosing rhabdomyolysis [25]. Physicians should pay more attention to the potential development of rhabdomyolysis in patients with continuous use of tocolytic therapy. In addition, some experts have suggested that basal and routine measurements of CK levels are unnecessary in asymptomatic patients [26].

### Ritodrine-induced acute liver injury

There are several publications describing the association between ritodrine and acute liver injury. One prospective study reported that ritodrine was rarely associated with liver function impairment, even when intravenous ritodrine was administered in 128 pregnant women [27]. In that study, the incidence of acute liver injury in singleton and multiple pregnancies was approximately 1.9% and 9.1%, respectively. The physiological mechanism of liver impairment induced by ritodrine has been presumed to be that beta-adrenergic agents may increase the metabolic response, which causes an overload of liver function. The authors concluded that although beta-adrenergic agonists may induce an associated metabolic response in a majority of women, the elevation of liver enzymes is still rare. Therefore, toxicity is probably a distinct side effect of ritodrine [28, 29]. In our case, impaired liver function was observed after the use of intravenous ritodrine for approximately one week. Liver function gradually improved after we discontinued the tocolytic agents. We considered that both ritodrine and ritodrine-induced rhabdomyolysis were the leading causes of liver damage.

### The synergic effects of atosiban and ritodrine

Atosiban is a synthetic peptide that competitively blocks the oxytocin receptor and prevents the action of oxytocin, resulting in uterine relaxation. Atosiban is the only tocolytic agent approved by the European Medicine Agency to stop premature labor, and it is currently used for acute tocolysis throughout Europe [30]. Tractocile (atosiban) was given intravenously with an initial bolus dose (6.75 mg/0.9 ml), followed by a high-dose continuous infusion for 3 h (loading infusion 300 micrograms/min) (Tractocile solution concentrate 37.5 mg/5 ml) and a lower dose with a subsequent infusion (100 micrograms/min) up to 45 h. The total treatment time did not surpass 48 h. Few side effects, including nausea, vomiting and headache, occurred [31].

There is limited evidence about the synergic effects of ritodrine and atosiban when used to stop preterm labor. Fu S et al. concluded that the extension of gestation in the combined treatment group was slightly shorter than that in the ritodrine-only group. However, the former group had a lower incidence of palpitations and adverse drug events. The authors suggested that the combination of ritodrine and atosiban can effectively treat premature labor and inhibit contractions with fewer adverse effects [32]. Wang R et al. found that the combined use of these tocolytic drugs could suppress uterine contractions faster and more effectively without increasing side effects, including nausea, headache, chest pain and tachycardia [33]. We combined both drugs for tocolysis and prolonged gestation by approximately two days without frequent uterine contractions, but side effects of ritodrine still occurred, such as acute liver injury and rhabdomyolysis.

## Conclusion

Mirror syndrome and ritodrine-induced pulmonary edema are difficult to identify clinically. In spite of limited evidence, we presume that ritodrine hydrochloride may exacerbate mirror syndrome in cases with chorioangiomas due to maternal fluid overload. Physicians should pay more attention to ultrasound examination of the placenta in patients developing mirror syndrome when using ritodrine. Prompt delivery is the best management for MS and ritodrine-induced pulmonary edema.

## Data Availability

All data generated or analysed during this study are included in this published article.

## Abbreviations

ALT: Alanine aminotransferase
AST: Aspartate aminotransferase
CK: Creatinine kinase
DM: Dystrophia myotonica
MS: Mirror syndrome
